# Simulation of the Thermodynamic Properties and Hydrophobicity of Polydimethylsiloxane Modified by Grafting Nano-SiO_2_ with Different Silane Coupling Agents

**DOI:** 10.3390/ma18102323

**Published:** 2025-05-16

**Authors:** Yuzhang Xie, Weiju Dai, Jingyi Yan, Zuhao Wang, Chao Tang

**Affiliations:** 1 College of Engineering and Technology, Southwest University, Chongqing 400700, China; 18671892009@163.com (Y.X.); swuwzh2155@163.com (Z.W.); 2 Electric Power Research Institute of Yunnan Power Grid, Kunming 650214, China; 15911530479@163.com (W.D.); yanjingyi1998@163.com (J.Y.)

**Keywords:** polydimethylsiloxane (PDMS), nano-SiO_2_, silane coupling agents, molecular dynamics

## Abstract

Polydimethylsiloxane (PDMS) with good hydrophobicity and nano-SiO_2_ with excellent thermal stability and mechanical properties are used as a composite coating for cellulose insulating paper in oil-immersed transformers, which effectively reduces the moisture generated by the thermal aging process, thus prolonging each transformer’s service life. This study employed molecular dynamics simulations to investigate the effects of surface-modified nano-SiO_2_ with different silane coupling agents (KH570 and KH151) on the thermodynamic properties and hydrophobicity of PDMS. Four groups of anhydrous models were constructed, namely, PDMS, P-SiO_2_, P-570, and P-151, as well as four corresponding groups of water-containing models: PDMS/H_2_O, P-SiO_2_/H_2_O, P-570/H_2_O, and P-151/H_2_O. The results demonstrate that incorporating silane-coupled nano-SiO_2_ into PDMS enhances mechanical properties, *FFV*, *CED*, *MSD*, diffusion coefficient, interaction energy, and hydrogen bond count, with KH570-grafted composites exhibiting optimal thermomechanical performance and hydrophobicity. At a temperature of 343 K, KH570 modification increased the bulk modulus and *CED* by 26.5% and 31.0%, respectively, while reducing the water molecular diffusion coefficient by 24.7% compared to that of unmodified PDMS/SiO_2_ composites. The extended KH570 chains occupy additional free volume, forming a larger steric hindrance layer, restricting molecular chain mobility, suppressing hydrogen bond formation, and establishing a low energy surface.

## 1. Introduction

Oil-immersed transformers, as one of the core components of power systems, play a pivotal role in the modern power industry, and the insulation performance of the internal oil–paper insulation system determines the life of the transformer and the safe and stable operation of the power system [1,2]. Inside the transformer, which is exposed to electromagnetic fields, a high-temperature environment, and the combined effects of mechanical stress, thermal aging leads to the degradation of the insulating paper’s performance. During aging, the oil–paper system generates moisture, a significant cause of insulation paper performance degradation [3]. Improving its thermodynamic and hydrophobic properties can extend its life.

Polydimethylsiloxane (PDMS) is a polymer consisting of organosiloxylated small molecules with good chemical stability and mechanical properties, excellent intrinsic hydrophobicity (water contact angle exceeding 110°), good deformation adaptability, and resistance to high temperatures, acids, and alkalis [4]. Cellulose insulating papers, as an irreplaceable part of oil-immersed transformers, have strict requirements for hydrophobicity and thermal stability. PDMS with these characteristics is particularly suitable for cellulose insulating paper and meets its performance requirements. It has been reported that PDMS can be applied to hydrophobic fabrics [5], deicing coatings [6], oil–water separation systems [7], etc. Stanton et al. constructed a superhydrophobic surface with PDMS on a textured substrate, and the resultant material surface achieved a static contact angle of 153.5° [8]. Xie et al. found that the chemical structure and roughness of the surface layer of a surface-fluoridated PDMS sheet were changed, thereby enhancing the hydrophobic properties and reducing the surface energy [9]. However, fluorinated solvents, which are usually required in the processing of fluorine-based materials, are not only harmful to the ozone layer but also potentially hazardous in the workplace due to bioaccumulation and long-range environmental transport [10,11]. Therefore, it is desirable to use non-fluorinated hydrophobic reagents to construct hydrophobic coated surfaces.

To optimize the performance, the introduction of nanoparticle (e.g., SiO_2_, Al_2_O_3_) modification has become a mainstream strategy [12,13], but the direct incorporation of high-surface-energy nanoparticles may cause nanoparticle agglomeration problems, which affect the overall insulating performance [14]. The silane coupling agent functions as a “molecular bridge” between nanoparticles and the polymer matrix (PDMS). This process can form an organic matrix–silane coupling agent–inorganic matrix bonding layer, thereby optimizing the dispersion and interfacial bonding strength of the nanoparticles and enhancing hydrophobicity [15]. Agrawal et al. proposed a novel method that utilizes fluorine-free silane coupling agents and copper oxide (CuO) nanoparticles applied to PDMS-coated cotton fabrics to fabricate a highly durable superhydrophobic material [16]. Tang et al. modified graphene oxide (GO) using a silane coupling agent doped into polydimethylsiloxane (PDMS) polymers and prepared novel coatings that showed significant enhancement in mechanical and thermal conductivity properties [17]. Chua et al. applied the silane coupling agent 3-aminopropyltriethoxysilane to surface functionalize multi-walled carbon nanotubes (MWNTs), investigating its impact on PDMS nanocomposite properties. The results demonstrated the effective enhancement of dynamic mechanical performance coefficients and thermal conductivity, alongside reduced electrical conductivity [18]. In this study, two silane coupling agents, γ-methacryloxypropyltrimethoxysilane (KH570) and vinyltrimethoxysilane (KH151), which are frequently employed for nanoparticle modification, were selected for comparative analysis. The molecular chain length of KH570 exceeds that of KH151, and this variation was utilized to examine the impact of varying silane coupling agent chain lengths on the thermodynamic properties and hydrophobicity of PDMS-modified materials.

Molecular dynamics simulation refers to the computational simulation of the spatial motion of atoms and molecules over time based on Newtonian mechanics. This approach is used to reveal the microstructure, thermodynamic properties, and kinetic processes of the system. The advent of computer technology has led to the widespread adoption of molecular dynamics simulation techniques in research owing to their advantages in examining the doping modification of the polymer matrix by nanoparticles at the microscopic level [19,20,21]. In this study, the properties of nano-SiO_2_@PDMS-modified cellulose insulating paper with different types of silane coupling agents were investigated based on molecular dynamics simulations using Materials Studio 7.0 software. A pure PDMS model, a PDMS model of doped surface-hydrogenated nano-SiO_2_, a PDMS model of doped surface-grafted KH570 nano-SiO_2_, and a PDMS model of doped surface-grafted KH151 nano-SiO_2_ were constructed. The mechanical properties, fractional free volume, cohesive energy density, mean square displacement, water molecule diffusion coefficient, hydrogen bonding, and interaction energy were calculated in each system to analyze the enhancement effect of nano-SiO_2_ as well as the grafting of different kinds of silane coupling agents on the thermodynamic and hydrophobic properties of PDMS matrix materials. The calculation results obtained from the simulations can provide micro-level data for practical applications related to PDMS while establishing a theoretical foundation for improving cellulose insulating paper’s hydrophobicity to delay aging.

## 2. Modeling and Simulations

PDMS exists in two forms, crystalline and amorphous zones. Relative to the crystalline zone, the amorphous zone is more loosely arranged with weaker intermolecular forces and is more unstable, so this study focuses on doping the amorphous zone of PDMS with nano-SiO_2_ modified with different types of silane coupling agents and constructing an amorphous cell model. However, the thermodynamic and hydrophobic properties of different kinds of silane coupling agent-grafted nano-SiO_2_ doped into PDMS are different, which necessitates investigation by constructing composite models with different variables separately.

### 2.1. Molecular Model Establishment

**Figure 1:**
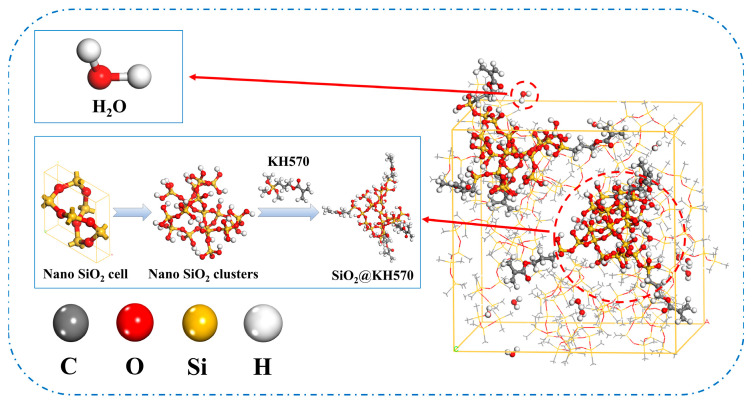
P-570/H_2_O model construction process.

**Figure 2:**
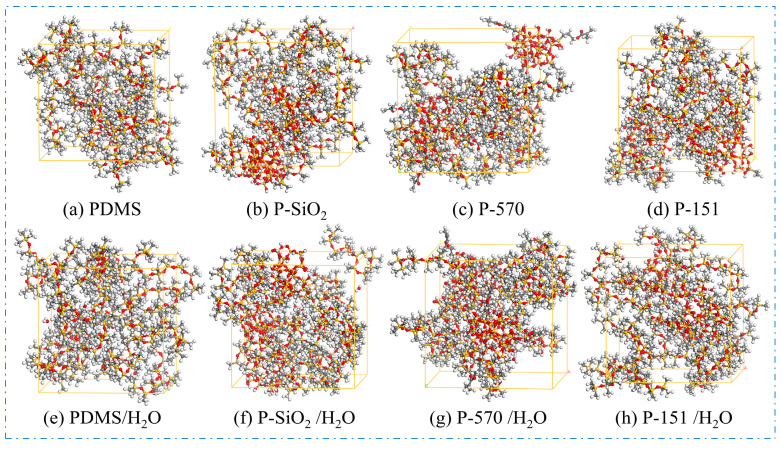
Molecular conformations of each model.

To investigate the effect of different silane coupling agents modifying SiO_2_ (KH151, KH570), focusing on its thermodynamic properties and hydrophobicity, four anhydrous composite models and four aqueous composite models were constructed. The four anhydrous composite models were PDMS, PDMS-doped surface-hydroxylated nano-SiO_2_ (P-SiO_2_), PDMS-doped surface-grafted KH570 (P-570), and PDMS-doped surface-grafted KH151 (P-151) nanosized SiO_2_. To facilitate the observation of the distribution of water molecules in the coatings, 15 water molecules were added to each of the above corresponding anhydrous models, and four water-containing composite models were obtained, which were named PDMS/H_2_O, P-SiO_2_/H_2_O, P-570/H_2_O, and P-151/H_2_O, respectively. The P-570/H_2_O model construction process is illustrated in Figure 1. The specific modeling process is outlined as follows:

- SiO_2_ nanoparticles with a 5 Å diameter were constructed and subjected to surface hydrogen termination. Subsequently, each nanoparticle was functionalized with four silane coupling agent molecules, achieving a grafting density of 12.5%. The modified nanostructures then underwent geometry optimization using the COMPASS force field.
- PDMS is a widely used hydrophobic material. Published reports indicate that in molecular simulations of polymeric systems, excessive polymer quantities or extended chain lengths can significantly prolong relaxation times. A degree of polymerization between 10 and 20 has been demonstrated to enable accurate simulations of composite material properties [22]. Consequently, this study adopts a polymerization degree of 10. Single-chain PDMS with this polymerization degree was subjected to geometry optimization using the COMPASS force field.
- Composite crystal cell models were constructed using the Amorphous Cell module. Eighteen PDMS molecular chains were combined with SiO_2_, SiO_2_@KH570, and SiO_2_@KH151 nanoparticles in the composite cell model with a controlled nanoparticle mass fraction of 30%, and the density was set to be 0.97 g/cm^3^. As shown in Figure 2, four anhydrous models and four water-containing models were constructed.

### 2.2. Simulation Details

The initial state of the composite model established through the amorphous region is relatively active, so the structure optimization of the established composite model is repeated many times to induce energy convergence until the energy cannot be lowered any more, that is, the structure optimization is completed. Then, to make the hole distribution of the cell closer to that of the real material, the optimized composite model is annealed for 10 cycles at 300 K~900 K using the NVT system, and after the annealing is completed, it is necessary to carry out an energy minimization treatment by selecting the model with the lowest energy to carry out the molecular dynamics simulation. After judging that the model is reasonable, the molecular dynamics simulation is carried out successively with 500 picoseconds (ps) of NVT and 500 ps of NPT, with the temperature set at 298 K, and the molecular dynamics information is collected at a frequency of once every 0.1 ps.

Among the commonly used force fields, the COMPASS force field is an all-atom force field that has found wide application in materials science and chemistry. It is applicable to organic molecules, inorganic materials, polymers, and interfacial systems, and it supports simulations from gas phase to condensed phase [22]. The Nose method is a technique that maintains a constant temperature during the simulation by introducing additional variables that simulate a thermal reservoir. The Berendsen method is a system that regulates pressure by adjusting the volume of the simulated cell according to the difference between the internal and target pressures. The Ewald method, an exact technique for calculating long-range interactions in periodic systems, divides the interaction term into two parts: real space and reciprocal space, while the atom-based method considers the dispersed interactions of atom pairs within a defined cutoff radius [23].

**Figure 3:**
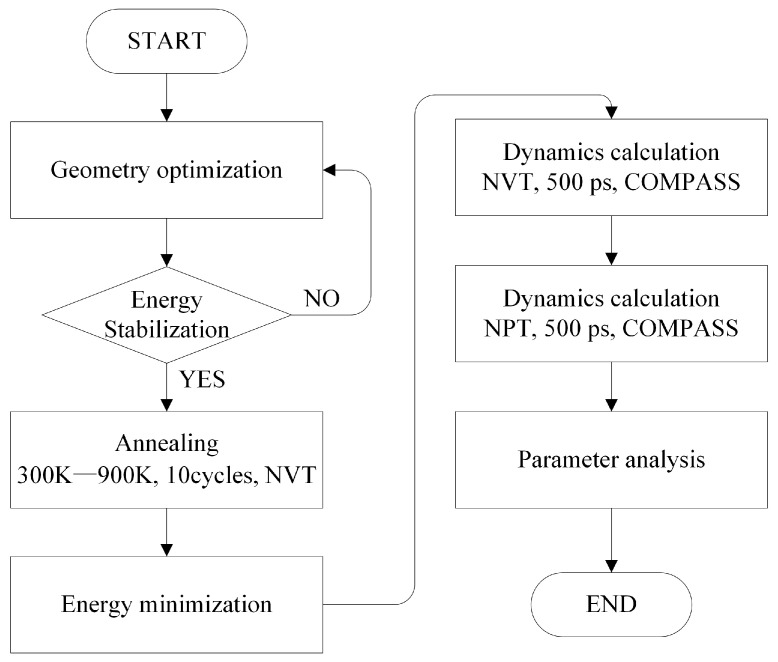
Molecular dynamics simulation flow chart.

Considering the combination of inorganic and organic molecular systems, the COMPASS force field is used throughout the simulation. The Nose method is used to control the temperature, and the Berendsen method is used to control the pressure. The atom-based method is used for van der Waals interaction and the Ewald method for electrostatic interaction. The simulation details are shown in Figure 3.

## 3. Analysis of Thermodynamic Properties

### 3.1. Mechanical Properties

For the PDMS composite models mentioned above, the elastic modulus *E*, shear modulus *G*, and bulk modulus *K* are key indicators of their mechanical properties [24]. Among them, the bulk modulus is the relationship between the bulk strain and the average stress of the material, reflecting the incompressibility under external forces; the shear modulus characterizes the material’s resistance to deformation under shear stress applied to the model, reflecting the model’s ability to resist the tangential stresses; and the modulus of elasticity is the ratio of the external stress applied to the model to the resulting strain, reflecting the model’s ability to resist deformation [25]. A small stress is applied to the composite model to simulate how it produces strain under stress, and the system’s stress–strain relationship follows Hooke’s law:

(1) $$\sigma_{i}=C_{ij}\varepsilon_{j}$$

where σ_i_ is the stress, C_ij_ is the matrix of elastic stiffness coefficients, and $\varepsilon_{j}$ is the strain tensor. The elastic modulus E, the shear modulus G, and the bulk modulus K of the composite material can be expressed in terms of the elastic constants λ and μ as follows:

(2) $$E=\mu \frac{3\lambda +2\mu }{\lambda +\mu }$$

(3) G=μ

(4) $$K=\lambda +\frac{2}{3}\mu$$

In the working environment within the actual transformer, the PDMS composites are subjected to significant stresses from external factors such as thermal stresses, electromagnetic forces, and mechanical vibrations, so it is necessary to enhance their modulus of elasticity E, shear modulus G, and bulk modulus K to extend the service life of the oil-immersed transformer. The following four anhydrous models were evaluated for mechanical properties: PDMS, P-SiO_2_, P-570, and P-151. To simulate the ambient temperature of actual transformer operation, the temperature was set to 343 K.

**Figure 4:**
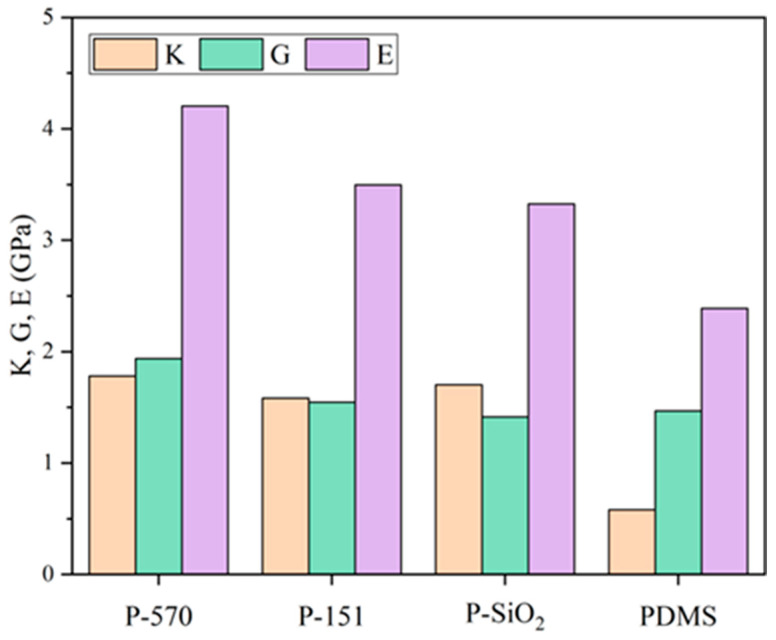
Mechanical properties of the models at 343 K.

As shown in Figure 4, the elastic modulus and bulk modulus of the doped nanoparticle composite system exhibit a marked increase compared to those of the pure PDMS system, with the shear modulus also demonstrating slight enhancement. This observation suggests that doping nano-SiO_2_ enhances the composites’ mechanical properties, which can be further improved by surface treatment with a silane coupling agent. Comparing the modification effects of the three groups of doped nanoparticle systems in terms of mechanical properties, the composite system of P-570 showed the best improvement in elastic modulus, shear modulus, and bulk modulus, and the mechanical properties were improved by 4.5%, 37.0%, and 26.5%, respectively, concerning the P-SiO_2_ system, which was attributed to the highest energy of interaction between the nano-SiO_2_ grafted with KH570 and the PDMS. Compared with that of KH151, the long oxidized heterozygous chain of KH570 not only formed a larger steric hindrance layer, which not only reduced nanoparticle agglomeration and contributed to the uniform distribution of stress but also provided a certain degree of toughness and mitigated brittle fracture due to stress concentration.

### 3.2. Fractional Free Volume

According to the free volume theory, free volume refers to intermolecular voids in materials, while occupied volume represents the space filled by molecular chains. The free volume creates dynamic spaces that facilitate molecular chain mobility. The ratio of the free volume of a polymer to its total volume, denoted as the fractional free volume (*FFV*), exerts a significant influence on the macroscopic material’s properties. This theoretical framework has become instrumental in analyzing the diffusion mechanisms and transport characteristics of polymeric materials [26]. The *FFV* is calculated using the following expression:

(5) $$FFV=\frac{V_{f}}{V_{o}+V_{f}}\times 100\%$$

where V_f_ denotes the free volume and V_O_ denotes the occupied volume.

**Figure 5:**
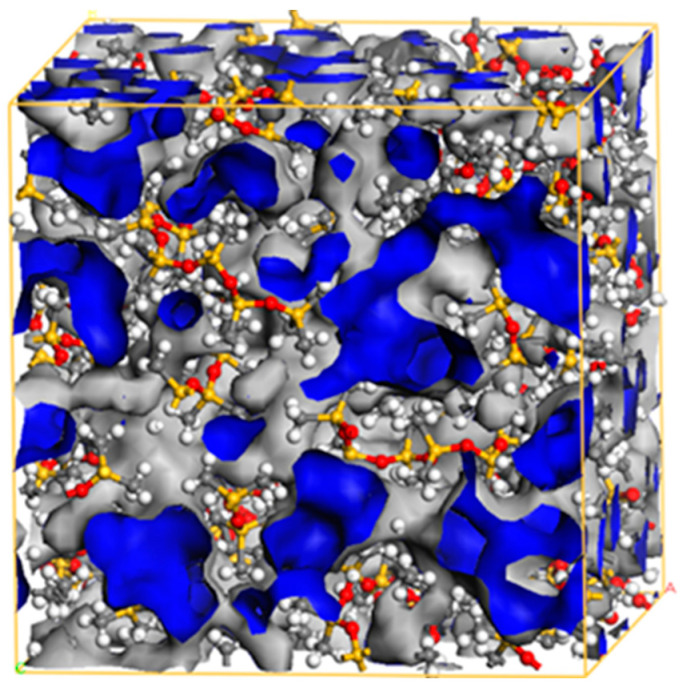
Schematic diagram of the *FFV* of the P-570 model.

The P-570 model serves as an example for constructing a model diagram of the *FFV* at a temperature of 343 K, as shown in Figure 5. Computational simulations were performed to calculate the *FFV* of four anhydrous models, PDMS, P-SiO_2_, P-570, and P-151, at varying temperatures, with the results listed in Table 1.

The larger the *FFV*, the more active the molecular mobility. As can be seen from Table 1, the free volume increases and the distribution becomes more disordered as the temperature increases. In the NPT system, the model exchanges energy with the outside world, and thermal expansion dominates. With increasing temperature, the tendency of molecular chain segments to move is further enhanced, reducing stacking efficiency and enlarging the free volume V_f_, while the size of the occupied volume V_O_ remains almost unchanged, increasing the *FFV*. This phenomenon is most pronounced in the P-SiO_2_ system. When the temperature changes from 323 K to 363 K, its *FFV* surges by 23.4%, reflecting the sensitivity of its rigid structure to thermal perturbation. As derived from Table 1, the two groups of composite models (P-570, P-151) grafted with silane coupling agents exhibit relatively lower *FFV* values. In particular, the volume fraction of the P-570 model decreases most significantly. This is because the surface modification of SiO_2_ with silane coupling agents enables tighter bonding between PDMS and nano-SiO_2_, enhancing the inhibition of chain movement and thereby reducing the *FFV*.

### 3.3. Cohesive Energy Density

Cohesive energy density (*CED*) refers to the energy needed to overcome intermolecular forces during the vaporization of a unit volume of condensed matter. It can be used to describe the interaction strength of a polymer and to evaluate its intermolecular interaction forces. The larger the *CED*, the more energy the polymer requires to overcome intermolecular forces and the more stable and difficult to destroy the molecules are [27]. The *CED* is calculated as follows:

(6) $$CED=\frac{\Delta H-RH}{V_{m}}=\frac{E_{coh}}{V_{m}}$$

In this equation, ΔH is the vaporization temperature, R is the gas constant, V_m_ is the molar volume, and E_coh_ is the cohesive energy. In polymer systems, internal non-bonding energy is divided into intermolecular and intramolecular components; the *CED* specifically reflects the intermolecular portion. The calculation of this energy can be derived from the principles of statistical mechanics as follows:

(7) $$E_{coh}=E_{inter-nonbond}=-\left(E_{VDW}+E_{coul}+E_{Hbond}\right)$$

**Figure 6:**
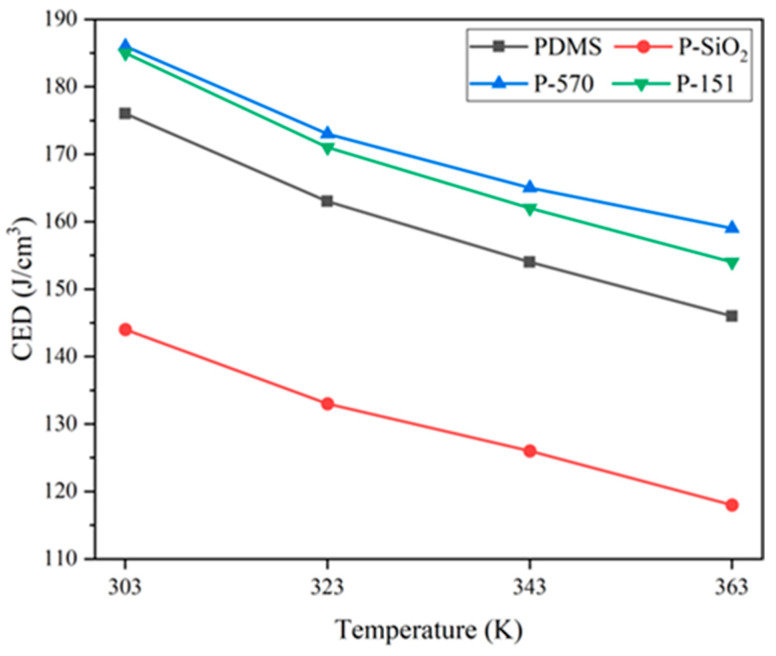
CED for each model at different simulation temperatures.

A simulation was conducted on four groups of anhydrous models, with temperatures ranging from 303 K to 363 K. Data were collected at 20 K intervals. The results of the *CED* with different temperatures for the various models are presented in Figure 6.

Figure 6 shows that the *CED* in all models decreases with rising temperature, attributed to enhanced molecular chain mobility and reduced internal compatibility under thermal excitation. The general trend of the *CED* of each model is as follows: P-570 > P-151 > PDMS > P-SiO_2_. The surface of nano-SiO_2_ without a grafted silane coupling agent is rich in hydroxyl groups, which are poorly compatible with the PDMS matrix, and the interfacial bonding is weak, weakening the intermolecular force at the interface and forming a certain void, which leads to a decrease in the *CED* of the system. The grafting of silane coupling agents (KH570, KH151) has been shown to strengthen interfacial bonding, improve nanoparticle dispersion, increase the effective filler–matrix contact area, and boost the system’s *CED*. Comparing the composite models of two different grafted silane coupling agents (P-570, P-151), the *CED* value of the P-570 model was the highest, which indicated that between the modified nanoparticles and the PDMS chains, the bonding was tighter and the molecular structure more stable.

### 3.4. Mean Square Displacement

**Figure 7:**
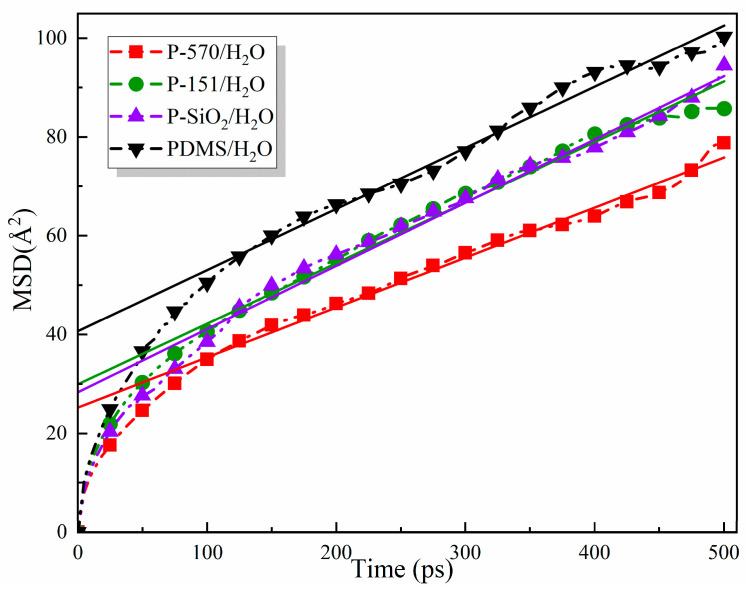
*MSD* curves and linear fit lines of each model at 343 K.

Chain motion serves as a key parameter for assessing material thermal stability. The mean square displacement (*MSD*), which characterizes chain motion, shows that an increased *MSD* value is indicative of a more pronounced chain motion, which consequently results in a diminished degree of material thermal stability [28,29]. Its calculation formula is as follows:

(8) $$MSD=\langle {\left|r_{i}\left(t\right)-r_{i}\left(0\right)\right|}^{2}\rangle$$

where r_i_(t) and r_i_(0) are the position vectors of atom i at time t and time 0, respectively. To investigate the overall movement of the center of mass of the molecular chains of each model, a total of 500 ps of mean square displacements were calculated for the four water-containing models, namely PDMS/H_2_O, P-SiO_2_/H_2_O, P-570/H_2_O, and P-151/H_2_O, at a temperature of 343 K. The results of these calculations are displayed in Figure 7.

As shown in Figure 7, under the simulated temperature of 343 K, the *MSD* values of the four models were as follows: PDMS/H_2_O ranged from 0 to 100 Å, P-SiO_2_/H_2_O ranged from 0 to 94 Å, P-151/H_2_O ranged from 0 to 85 Å, and P-570/H_2_O ranged from 0 to 78 Å. These results indicate that the thermal stability of the four water-containing models follows the order P-570/H_2_O > P-151/H_2_O > P-SiO_2_/H_2_O > PDMS/H_2_O, and the incorporation of KH570-grafted SiO_2_ nanoparticles is demonstrated to exert the most significant influence on the *MSD* of PDMS. This phenomenon can be attributed to the presence of SiO_2_ nanoparticles, which have been observed to fill the interstices between the molecular chains of PDMS, thereby occupying the available volume within the system. This results in a compact arrangement of the internal molecules and an enhancement in their interactions. The incorporation of a silane coupling agent serves to enhance the bond strength between the PDMS chains and SiO_2_ nanoparticles, thereby increasing the interfacial bonding strength. This modification has the potential to effectively reduce the chain movement of the system and enhance its thermal stability under elevated temperature and pressure conditions. A comparison of the two different silane coupling agents reveals that the long-chain structure of KH570 significantly reduces the *MSD* of the system compared with KH151. This reduction can be attributed to the flexible chain segments of KH570, which physically entangle with the PDMS substrate to build a dense interfacial network. This network effectively restricts the molecular chain movement. In contrast, KH151 relies exclusively on chemical bonding and lacks the ability to engage in physical entanglement. The interfacial bonding is looser, which leads to the intensification of molecular chain movement and lower thermal stability than that of the P-570/H_2_O composite model.

## 4. Hydrophobicity Analysis

### 4.1. Diffusion Coefficient

Water molecules represent small molecules in the whole composite system, and their movement in the system can be characterized by the diffusion coefficient. From Einstein’s relation, there exists a corresponding connection between the *MSD* and the diffusion coefficient, and one-sixth of the slope of the curve of the *MSD* is the value of the diffusion coefficient [30]; the relation is as follows:

(9) $$D=\frac{1}{6N}\underset{t\to \infty }{lim}\frac{d}{dt}\sum_{i=1}^{N}\langle {\left|r_{i}\left(t\right)-r_{i}\left(0\right)\right|}^{2}\rangle$$

Consequently, the diffusion coefficients of H_2_O in the composite system can be obtained by fitting the *MSD* curves of the four water-containing systems. The calculated results are presented in Table 2.

The diffusion coefficients of all models exhibited a significant increase with increasing temperature (303 K~343 K), a phenomenon that adhered to Arrhenius’ law. This observation signifies that an elevated temperature led to a weakening of the internal binding effect of the matrix material, thereby accelerating the diffusion of water molecules. The larger the diffusion coefficient is, the worse the hydrophobic performance is. When the simulated temperature is 343 K, the diffusion coefficients of the four models are ranked as follows: P-570/H_2_O > P-151/H_2_O > PDMS/H_2_O > P-SiO_2_/H_2_O. The introduction of nano-SiO_2_ reduces the free volume by physical filling, and its surface is rich in hydroxyl groups that can form hydrogen bonds with PDMS chains, which limits the migration of water molecules to a certain extent. However, as the temperature rises from 323 K to 343 K, the hydrogen bonds become thermally unstable, leading to a weakening of the interfacial interaction. This phenomenon is evidenced by a substantial increase in the diffusion coefficient of water molecules, which exceeds that of the composite model of PDMS/H_2_O. The introduction of a silane coupling agent significantly inhibited the diffusion of water molecules inside the system, and the modification effect of KH570 was best, with the water molecule diffusion coefficient decreasing by 22.6% at 343 K compared with that of the PDMS/H_2_O composite model.

### 4.2. Interaction Energy

The interaction energy is a critical parameter for characterizing the binding state of substances, with negative interaction energy denoting the propensity for spontaneous adsorption of the system [31]. In this study, three composite models of SiO_2_ nanoparticles and their surface modification were constructed based on molecular dynamics, with their interaction energy with water molecules quantified. The temperature parameter of the simulation process was set to 343 K. The interaction energy E between the systems was defined as follows:

(10) $$E=E_{total}-\left(E_{H_{2}O}+E_{mixture}\right)$$

where E_total_ is the total energy of the system, E_H2O_ is the molar internal energy of the water molecules in the system, and E_mixture_ is the molar internal energy of the substances in the system other than water molecules. In this paper, the interaction energies for three water-containing composite models are quantified, with corresponding values summarized in Table 3.

The interaction energy reflects the system’s affinity for water molecules. A higher absolute value of (negative) interaction energy indicates a stronger attraction and lower hydrophobicity. As shown in Table 3, the P-SiO_2_/H_2_O composite model exhibits the most negative interaction energy of −100.57 kJ·mol^−1^ with water molecules, significantly exceeding the energy of silane coupling agent-grafted models (P-570/H_2_O and P-151/H_2_O). This result demonstrates the poorest hydrophobic performance. In contrast, the P-570/H_2_O composite shows a substantially reduced interaction energy of −38.38 kJ·mol^−1^, representing a 61.8% decrease compared to P-SiO_2_/H_2_O and a 30.8% decrease relative to P-151/H_2_O, reflecting the superior hydrophobicity. Therefore, we conclude that PDMS composites doped with nano-SiO_2_ and grafted with KH570 achieve optimal hydrophobic performance, as evidenced by their minimized interaction energy with water molecules.

### 4.3. Hydrogen Bonding

**Figure 8:**
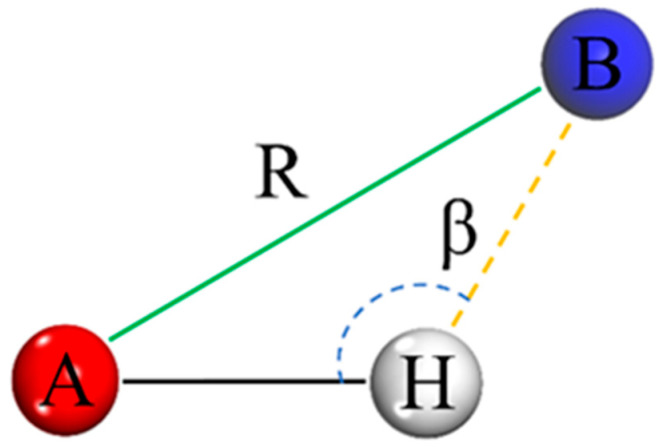
Definition of hydrogen bond in molecular simulation.

Hydrogen bonding is a special interaction between strong and weak chemical bonds with bonding energies similar to van der Waals forces. If the surface of a material contains a large number of hydrogen bond donors (e.g., hydroxyl groups, amino groups) or acceptors (e.g., O, N, and F atoms), water molecules can easily bond with them through hydrogen bonding to form a hydration layer, which is hydrophilic; on the contrary, the smaller the number of hydrogen bonds is, the harder it is for water molecules to be adsorbed stably, and the more hydrophobic the material is. Hydrogen bonds (A-H…B) can be determined through two criteria: geometry and energy [32]. In this paper, the geometrical criterion is chosen to determine the hydrogen bond (A-H…B), and the principle is shown in Figure 8, where the constraints for the formation of the hydrogen bond are set to have a distance R of 3 Å between the atoms of A and B and a bond angle β of 120°. For the number of hydrogen bonds formed between water molecules and the composite system for the three water-containing models at temperatures ranging from 303 K to 343 K, a Perl script was imported to calculate the number of hydrogen bonds in each model, and for the convenience of the subsequent analyses, the results of the calculations were taken as the total number of hydrogen bonds in the 100 output frames of the trajectory, as shown in Table 4.

With the increase in temperature, the number of hydrogen bonds in each system showed a decreasing trend in general, which was mainly considered to indicate that the molecular thermal motion was intensified after the energy of the system was increased, making it difficult to maintain a stable hydrogen bonding network, and the hydrogen bond energy was low, making it easy to be destabilized at high temperatures. According to Table 4, hydrogen bond totals across the three models demonstrated the following ranking: P-SiO_2_/H_2_O > P-151/H_2_O > P-570/H_2_O. The above findings demonstrated a decrease in the number of hydrogen bonds for the two composite models grafted with silane coupling agents, KH570 and KH151, when compared with the P-SiO_2_/H_2_O model. The percentage of decrease was 35.3% and 17.5%, respectively, under the condition of 343 K. Obviously, the composite models grafted with silane coupling agents KH570 and KH151 show a decreasing trend of hydrogen bonding. Incorporating silane-coupled nano-SiO_2_ effectively reduces hydrogen bonds with water molecules and enhances hydrophobicity. This is because the unmodified silica nanoparticles are rich in hydroxyl groups on the surface, which makes it easy to form hydrogen bonds with water molecules, and they have poor hydrophobicity, whereas the grafted silane coupling agent nanoparticles not only have a few hydroxyl groups on the surface but also inhibit the formation of hydrogen bonds due to the spatial resistance effect brought by the long chains. In addition, grafting the silane coupling agent allows the nanoparticles to form a more uniformly distributed micro-nanostructure in the matrix material, which enhances the hydrophobicity through physical roughness. At the same simulation temperature, the number of hydrogen bonds of the P-570/H_2_O composite model is significantly less than that of the P-151/H_2_O composite model, which is attributed to the fact that its long chains not only cover the hydroxyl groups on the silica surface more effectively and inhibit the formation of hydrogen bonds but also form a low-energy surface through the intermolecular arrangement.

### 4.4. Performance Comparison

From the above parameter analysis, it can be seen that KH570 has the best performance considering the thermodynamic and hydrophobic properties of PDMS-modified materials. As shown in Table 5, we compare the modification scheme of P-570 with a list of schemes mentioned in the literature and cited in the introduction, analyze their respective strengths and limitations, and find that the PDMS modification scheme of P-570 is more suitable for cellulose insulating paper.

## 5. Conclusions

In this paper, the thermodynamic properties and hydrophobicity of nano-SiO_2_ modified polydimethylsiloxane grafted with different silane coupling agents (KH570, KH151) were investigated using a molecular simulation technique by modeling performance parameters such as *FFV*, *CED*, *MSD*, diffusion coefficient, and interaction energy. The conclusions are summarized below:

1. The grafting of silane coupling agents onto SiO_2_ nanoparticle surfaces effectively enhances interfacial bond strength, reduces *FFV*, inhibits chain movement, and mitigates agglomeration, promoting uniform stress distribution and significantly enhancing the composites’ thermomechanical properties. The parameters are manifested in the enhancement of mechanical modulus and cohesive energy density, as well as the decrease in *FFV* and *MSD* values.
2. Silane coupling agent grafting on nano-SiO_2_ effectively reduces hydrogen bonds between the PDMS matrix material and water molecules, forming a uniform micro-nanostructure, lowering the diffusion coefficient, and decreasing interaction energy, thereby enhancing composite hydrophobicity.
3. Grafting different silane coupling agents variably influenced the composites’ thermodynamic and hydrophobic properties. KH570 showed the best enhancement effect on the thermomechanical and hydrophobic properties of the composites, with its system exhibiting 4.5%, 37.0%, 26.5%, and 31.0% increases in elastic, shear, and bulk moduli and *CED* compared to the pure SiO_2_ composite model at 343 K simulation. Meanwhile, the *FFV*, *MSD*, diffusion coefficient, interaction energy, and hydrogen bonds decreased by 9.1%, 16.6%, 24.7%, 61.8%, and 35.3%, respectively. The reason is that the flexible long chain of KH570 not only occupies more free volume but also can become physically entangled with the PDMS molecular chain, which restricts the molecular chain movement. In addition, the flexible long chains can form a larger steric hindrance layer, which inhibits the formation of hydrogen bonds and constructs a low-energy surface.

## Figures and Tables

**Table 1:** Fractional free volume for different models at different temperatures.

Model	Parameter	Temperature		
		323 K	343 K	363 K
PDMS	V_F_/Å^3^	6499.96	6872.70	7230.11
	V_O_/Å^3^	17,726.93	17,649.50	17,606.21
	FFV/%	26.83	28.03	29.11
P-SiO_2_	V_F_/Å^3^	6059.03	7634.04	8115.44
	V_O_/Å^3^	20,823.32	20,494.44	20,846.63
	FFV/%	22.54	27.14	28.02
P-570	V_F_/Å^3^	6515.75	7094.46	7985.02
	V_O_/Å^3^	21,695.82	21,677.88	21,333.76
	FFV/%	23.10	24.66	27.24
P-151	V_F_/Å^3^	6200.29	7041.56	7606.01
	V_O_/Å^3^	19,661.20	19,571.17	19,497.76
	FFV/%	23.97	26.46	28.06

**Table 2:** Diffusion coefficients for each water-containing composite model.

Model	Diffusion Coefficient (10^−8^ cm^2^/s)		
	303 K	323 K	343 K
PDMS/H_2_O	134.6	168.6	198.7
P-SiO_2_/H_2_O	116.6	168.2	204.0
P-570/H_2_O	110.2	124.8	153.7
P-151/H_2_O	111.8	136.9	177.1

**Table 3:** The interaction energy of water molecules and complexes.

Model	E_total_ (kJ·mol^−1^)	E_H2O_ (kJ·mol^−1^)	E_mixture_ (kJ·mol^−1^)	E (kJ·mol^−1^)
P-SiO_2_/H_2_O	−21,470.26	−33.13	−21,336.56	−100.57
P-570/H_2_O	−19,564.68	−44.51	−19,481.79	−38.38
P-151/H_2_O	−18,948.10	−47.95	−18,844.72	−55.43

**Table 4:** Hydrogen bond totals between water molecules and complexes.

Model	Number of Hydrogen Bonds		
	303 K	323 K	343 K
P-SiO_2_/H_2_O	4149	3704	3436
P-570/H_2_O	2807	2554	2223
P-151/H_2_O	3519	3013	2834

**Table 5:** Strength and limitation analysis of different PDMS modification schemes.

Case	Research Method	Strengths and Limitations
P-570	Molecular dynamics simulation	Excellent thermomechanical performance and hydrophobicity.
PDMS [8]	Experiment, templating technique	Excellent hydrophobicity, unknown thermodynamic properties, templating technique is not suitable for the preparation of hydrophobic coatings for insulating paper.
Surface fluoridated PDMS [9]	Experiment, direct fluorination	High hydrophobicity, partially conductive, low surface energy, but the use of fluorine-based materials is harmful to the environment.
CuO@PDMS [18]	Experiment	Highly hydrophobic and antibacterial, high durability, but poor chemical stability and insulating properties of nano-CuO.
GO@PDMS [18]	Experiment	Excellent thermomechanical properties with erosion resistance, but more expensive and less insulating than nano-SiO_2_.
MWNT@PDMS [18]	Experiment	Excellent thermodynamic and electrical properties, but the cost of MWNTs is high, and the preparation process of functionalized modification is complicated.

## Data Availability

The original contributions presented in this study are included in the article. Further inquiries can be directed to the corresponding author.
